# An Exploratory Pilot Study with Plasma Protein Signatures Associated with Response of Patients with Depression to Antidepressant Treatment for 10 Weeks

**DOI:** 10.3390/biomedicines8110455

**Published:** 2020-10-28

**Authors:** Eun Young Kim, Hee-Sung Ahn, Min Young Lee, Jiyoung Yu, Jeonghun Yeom, Hwangkyo Jeong, Hophil Min, Hyun Jeong Lee, Kyunggon Kim, Yong Min Ahn

**Affiliations:** 1 Mental Health Center, Seoul National University Health Care Center, 1 Gwanak-ro, Gwanak-gu, Seoul 08826, Korea; ey00@snu.ac.kr; 2 Department of Human Systems Medicine, Seoul National University College of Medicine, 101 Daehak-ro, Jongno-gu, Seoul 03080, Korea; 3 Asan Institute for Life Sciences, Asan Medical Center, Seoul 05505, Korea; zaulim3@gmail.com (H.-S.A.); yujiyoung202@gmail.com (J.Y.); 4 Institute for Systems Biology, Seattle, Washington, DC 98109, USA; minyoung.lee@gmail.com; 5 Convergence Medicine Research Center, Asan Institute for Life Sciences, Asan Medical Center, Seoul 05505, Korea; nature8309@gmail.com; 6 Department of Biomedical Sciences, University of Ulsan College of Medicine, Seoul 05505, Korea; hkyo723@naver.com; 7 Doping Control Center, Korea Institute of Science and Technology, Hwarang-ro 14-gil 5, Seongbuk-gu, Seoul 02792, Korea; mhophil@kist.re.kr; 8 Department of Psychiatry & Behavioral Science, National Cancer Center, Ilsandong-gu, Goyang-si, Gyeonggi-do 10408, Korea; hjlee.np@gmail.com; 9 Division of Cancer Control & Policy, National Cancer Control Institute, National Cancer Center, Ilsandong-gu, Goyang-si, Gyeonggi-do 10408, Korea; 10 Clinical Proteomics Core Laboratory, Convergence Medicine Research Center, Asan Medical Center, Seoul 05505, Korea; 11 Bio-Medical Institute of Technology, Asan Medical Center, Seoul 05505, Korea; 12 Department of Neuropsychiatry, Seoul National University Hospital, 101 Daehak-ro, Jongno-gu, Seoul 03080, Korea; 13 Department of Psychiatry and Behavioral Science, Institute of Human Behavioral Medicine, Seoul National University College of Medicine, 101 Daehak-ro, Jongno-gu, Seoul 03080, Korea

**Keywords:** major depressive disorder, longitudinal study, LC-MS/MS, plasma protein biomarker, drug response monitoring, multiple reaction monitoring

## Abstract

Major depressive disorder (MDD) is a leading cause of global disability with a chronic and recurrent course. Recognition of biological markers that could predict and monitor response to drug treatment could personalize clinical decision-making, minimize unnecessary drug exposure, and achieve better outcomes. Four longitudinal plasma samples were collected from each of ten patients with MDD treated with antidepressants for 10 weeks. Plasma proteins were analyzed qualitatively and quantitatively with a nanoflow LC−MS/MS technique. Of 1153 proteins identified in the 40 longitudinal plasma samples, 37 proteins were significantly associated with response/time and clustered into six according to time and response by the linear mixed model. Among them, three early-drug response markers (PHOX2B, SH3BGRL3, and YWHAE) detectable within one week were verified by liquid chromatography-multiple reaction monitoring/mass spectrometry (LC-MRM/MS) in the well-controlled 24 patients. In addition, 11 proteins correlated significantly with two or more psychiatric measurement indices. This pilot study might be useful in finding protein marker candidates that can monitor response to antidepressant treatment during follow-up visits within 10 weeks after the baseline visit.

## 1. Introduction

Major depressive disorder (MDD) is one of the leading causes of disability worldwide [1], with a high prevalence among individuals of all ages and races [2]. MDD is a chronic condition with a high recurrence rate with a full recovery rate of only 20% and 80% of recovered patients experiencing at least one relapse in their entire life [3]. Antidepressants have long been used in the acute and long-term treatment of MDD, with selective serotonin reuptake inhibitors (SSRIs) being the first-line antidepressants. The process of selecting an antidepressant agent is primarily prescribed based on trial and error. Patients with poor efficacy of the initial course of medication for at least 4–6 weeks require alternative therapeutic strategies, containing changing within and between classes of antidepressants. Unfortunately, the treatment outcomes from antidepressants are discouraging. About 50% of patients enrolled in the Sequenced Treatment Alternative to Relieve Depression (STAR*D) study failed to respond to standard SSRI treatment, and only about 30% experienced complete remission in response to the first antidepressant used [4]. After unsuccessful treatment for MDD patients with a SSRI, the choice of a second drug is important for remission [5]. Biomarkers for response to antidepressant treatment can reduce the time to symptom relief and costs, minimize unnecessary drug exposure, and improve patient outcomes.

Proteomics, the quantitative analysis of all proteins expressed in samples, is a powerful tool for identifying novel molecular biomarkers and enables the detection of molecular signatures reflecting multiple biological pathways involved in response to treatment in patients with MDD [6]. Proteomic analysis of peripheral body fluids, such as blood plasma and serum, may not only enable prediction of response to treatment in clinical practice, but also assist in monitoring drug activity during early stages of clinical trials. To date, however, there have been few proteomic analyses of peripheral blood samples that can predict response to antidepressant treatment [7,8]. A previous liquid chromatography tandem mass spectrometry (LC-MS/MS) analysis found that several plasma proteins might be potential biomarkers for the prediction of antidepressant response over a 6-week treatment period [7]. A multiplex immunoassay testing of up to 258 blood-based markers related to immune, endocrine, and metabolic mechanisms identified 9 markers as potential pre-treatment biomarkers associated with antidepressants treatment response [8].

Longitudinal data are commonly used in biomedical studies [9,10]. In statistical analyses, mixed-effect models (MEMs) [11] and generalized estimating equations (GEEs) [12] are widely applied. To further elaborate, MEM is a subject-level approach that could employ random effects to acquire a between-subjects variable by considering the correlations with observations from the same subject based on the full-likelihood method. Conversely, GEE is a population-level model that relies on a partial-likelihood function. In this study, repeated drug efficacy measurements (baseline and follow-up visits after treatment) were performed on the surrogate plasma protein over time for each patient, with the subject of interest being some of the time-varying changes. In proteomic studies, the MEM method is more popularly used than the GEE method [13,14,15]. This is because after fixing the desired effect, it is possible to estimate by measuring a random effect of the technical or biological repeated measurement with actual MS [16,17]. In addition, it is technically easier to reflect the variance of any effect on repeated measurements of the same sample after more than two times of MS. Conversely, GEE is robust to the misspecification of correlation structure using quasi likelihood, and many modified variance estimation methods for small samples have been developed [18]. We identified biological implications primarily with the results of the analysis with linear mixed model (LMM) and compared the results after performing with the same data with GEE.

In this preliminary study, LC–MS/MS profiling was performed to identify candidate blood-based protein biomarkers that could monitor early (0–1 week), mid (1–4 week), or late (4–10 week) response to antidepressants, before and after their administration. This study also assessed whether changes in plasma protein concentrations after antidepressant treatment were associated with changes in the severity of depressive symptoms. Blood samples were collected at four time points during the 10-week treatment of ten depressed patients, five responders, and five non-responders, who were taking escitalopram. Plasma proteins were profiled, as were differences in protein abundance between the two groups. Unlike biomarker studies that don’t take into account the time of disease occurrence [19,20,21,22], this study attempted to identify more reliable candidate biomarkers by time-dependent longitudinal changes in the plasma proteome of these patients. Furthermore, the identified biomarkers predicting early-drug response were validated in 19 responders and five non-responders by the liquid chromatography-multiple reaction monitoring/mass spectrometry (LC-MRM/MS) technique. In addition, significant markers were identified assessing the correlation between protein concentrations, as determined by molecular diagnostic techniques, and psychological parameters.

## 2. Materials and Methods

### 2.1. Study Subjects

Since MADRS score is regarded as the criterion for determining response to drug administration, plasma samples were collected from ten patients with MDD who participated in a clinical trial testing the efficacy and safety of escitalopram dose escalation at Seoul National University Hospital, Seoul, Republic of Korea, from February 2013 to February 2016 [23]. All the participants are Korean patients. The trial included two phases: open-label treatment for 4 weeks with a standard dose (10–20 mg/day) of escitalopram, followed by randomized, double-blinded treatment for 6 weeks with 20 mg/day or 30 mg/day escitalopram. Patients aged 18–65 years with a primary diagnosis of MDD, as defined by the Diagnostic and Statistical Manual of Mental Disorders, 4th edition (text revision), were included. All patients had a total MADRS score ≥ 18 at initial screening and baseline visits. Subjects were excluded if they experienced hypersensitivity to escitalopram, had received any psychoactive medications such as antipsychotics, mood stabilizers, or selective monoamine oxidase inhibitors, had symptoms of depression and were deemed resistant to two or more antidepressant treatments, had psychiatric disorders other than MDD or a prior history of psychiatric disorders, such as manic or hypomanic episodes, schizophrenia, schizoaffective disorder, or substance abuse disorder, were at significant risk of suicide, as evaluated by an investigator or with score of ≥ 5 on item 10 of MADRS, or had a history of neurologic disorders or medically unstable conditions (e.g., renal or hepatic impairment, or cardiovascular, pulmonary, or gastrointestinal disorders). Of the patients who entered the clinical trial, five responders (1 male and 4 females) and five non-responders (1 male and 4 females) were selected, from each of whom plasma samples were obtained at four time points: baseline, week 1, week 4 (randomization), and week 10 (6 weeks after randomization) for proteomic analysis. An additional 24 patients were selected, 19 responders and five non-responders, from each of whom plasma samples were obtained at baseline and at week 1. The primary efficacy outcome was a change in total MADRS score. Response was defined as ≥50% reduction in baseline MADRS score after 4 and 10 weeks of treatment. None of these patients were taking medication that could alter the blood levels of relevant factors, such as nonsteroidal anti-inflammatory agents or steroids, and none had any acute or chronic diseases, such as cardiovascular disease, pulmonary disease, hypertension, endocrine abnormalities, rheumatic diseases, or cerebrovascular disease. The study protocol was approved by the Institutional Review Board of Seoul National University Hospital (Number: 1008-116-329, approved on 2 December 2010). The study was performed in accordance with the ethical principles stated in the Declaration of Helsinki and the International Conference on Harmonization Good Clinical Practice guidelines. All patients provided written informed consent and were free to discontinue the study at any time.

### 2.2. Blood Collection and Plasma Preparation

Plasma was prepared as suggested by the Human Proteome Organization Plasma Proteome Project. Blood samples (3 mL) were collected into ethylenediaminetetraacetic acid-containing tubes at baseline, week 1, week 4 (randomization), and week 10 (post-randomization week 6), and the blood samples were obtained from subjects after an overnight fast (at least 12 h) from 9:30 to 11:30 AM. Blood samples centrifuged at 2000× *g* for 15 min at room temperature (RT) immediately after sample collection. Plasma was transferred to 0.5 mL tubes and frozen within 20 min after centrifugation. Then, the samples were placed on ice and transported to the laboratory and immediately frozen at –80 °C until assayed.

### 2.3. Plasma Manipulation and Digestion

Plasma samples were sequentially subjected to high abundant plasma protein depletion and trypsin/Lys-C digestion. To remove the 14 most abundant plasma proteins (albumin, IgA, IgG, IgM, α1-antitrypsin, α1-acid glycoprotein, apolipoprotein A1, apolipoprotein A2, complement C3, transferrin, α2-macroglobulin, transthyretin, haptoglobin, and fibrinogen), a 40 μL aliquot of plasma diluted 4-fold with a proprietary “Buffer A” was injected into a MARS14 depletion column (Agilent Technology, Palo Alto, CA, USA) on a binary HPLC system (20A Prominence, Shimadzu, Tokyo, Japan). The unbound fraction was buffer-exchanged into 8 M urea in 50 mM Tris (pH 8), concentrated to approximately 50 μL by ultrafiltration using a Vivaspin 500 3 kDa cutoff filter (Sartorius, Goettingen, Germany), and then transferred to a new filter unit (Nanosep, 30 kDa; Pall Corporation, NY, USA). A 200 μL aliquot of 8 M urea in 50 mM Tris (pH 8.5) was added, and the mixture was centrifuged at 14,000× *g* for 15 min, with the procedure repeated twice. The flow-through from the collection tube was discarded, 100 µL of 0.05 M iodoacetamide solution was added, and the preparation was mixed at 600 rpm in a thermo-mixer for 1 min and incubated without mixing for 20 min. The filter units were centrifuged at 14,000× *g* for 10 min; 100 µL of 8 M urea in 50 mM Tris (pH 8.5) was added, and the filter units were again centrifuged at 14,000× *g* for 15 min, with this step repeated twice. A 100 µL aliquot of 0.05 M ammonium bicarbonate was added to the filter unit, and the unit was centrifuged at 14,000× *g* for 10 min, with this step also repeated twice. A 40 µL aliquot of 0.05 M ammonium bicarbonate containing 2.5 µg Lys-C/trypsin was added, and the preparation was mixed at 600 rpm in a thermo-mixer for 1 min. The units were incubated in a wet chamber at 37 °C for 12 h and transferred to new collection tubes. The filter units were centrifuged at 14,000× *g* for 10 min, 40 µL of 0.5 M NaCl was added, and the filter units were again centrifuged at 14,000× *g* for 10 min. The digestion reaction was stopped by the addition of formic acid to a final concentration of 0.3%. The peptide mixture was desalted with a Sep Pak C-18 cartridge (Waters, Milford, MA, USA), lyophilized with a cold trap (CentriVap Cold Traps, Labconco, Kansas City, MO, USA), and stored at −80 °C until used.

### 2.4. Nano-LC-ESI-MS/MS Analysis

Peptides were separated using a Dionex UltiMate 3000 RSLCnano system (Thermo Fisher Scientific, Waltham, MA, USA). Tryptic peptides from a bead column were reconstituted in 0.1% formic acid and separated on a 50 cm Easy-Spray column with a 75 μm inner diameter packed with 2 μm C18 resin (Thermo Fisher Scientific) over 200 min (250 nL/min). The column was developed using a 0–45% acetonitrile gradient in 0.1% formic acid and 5% DMSO at 50 °C. The LC was coupled to a Q Exactive mass spectrometer with a nano-ESI source. Mass spectra were acquired in a data-dependent mode with an automatic switch between a full scan and 20 data-dependent MS/MS scans. The target value for the full scan MS spectra was 3,000,000, with a maximum injection time of 120 ms and a resolution of 70,000 at m/z 400. Repeated peptides were dynamically excluded for 20 s. All MS data have been deposited in the PRIDE archive (www.ebi.ac.uk/pride/archive/projects/PXD017211) under Project PXD017211 [24].

### 2.5. Database Searching and Label-free Quantification

The acquired MS/MS spectra were searched using the SequestHT on Proteome discoverer (version 2.2, Thermo Fisher Scientific) against the SwissProt human database (May 2017). The search parameters were set as default including cysteine carbamidomethylation as a fixed modification, and N-terminal acetylation and methionine oxidation as variable modifications with two miscleavages. Peptides were identified based on a search with an initial mass deviation of the precursor ion of up to 10 ppm, with the allowed fragment mass deviation set to 20 ppm. When assigning proteins to peptides, both unique and razor peptides were used. Label-free quantitation (LFQ) was performed using peak intensity for unique peptides of each protein [25].

### 2.6. Analysis of Public Microarray Data

We downloaded the gene expression profile data (series accession number: GSE146446 [26] and GSE45468 [27]) in the Gene Expression Omnibus database for using Biobase and GEOquery package in R. Both data used the GPL570 platform (Affymetrix Human Genome U133 Plus 2.0 Array; Agilent Technologies, Palo Alto, CA, USA). We found the Affymetrix probe IDs by searching for the gene name. Then, subsequent statistical analysis was performed using the gene expression level value of each gene.

### 2.7. Batch Mean-Centering Correction, Missing Data Imputation, and Normalization

Three batches were prepared, with batch 1 consisting of S15 (non-responders) and S29 (responders); batch 2 of S54 (non-responders) and S52 (responders); and batch 3 of S6 (non-responders), S11 (non-responders), S32 (responders), S34 (non-responders), S38 (responders), and S46 (responders), based on sample preparation date [28]. Mean-centering correction per protein was applied to raw data from 104 LC-MS/MS analyses to avoid the batch effect [29,30].

Then, missing data imputation was performed. Of 316 quantified proteins measured at one time in each individual sample, 180 were completely quantified, whereas missing data for the remaining 136 proteins were determined by a local least-squares imputation method [31]. Using this method, the 180 completely quantified proteins were clustered into 15 groups by Pearson’s correlation analysis, and missing values were estimated by a linear optimal combination of the 15 selected clusters.

These data were normalized relative to endogenous normalizing proteins without spike-in standards [32]. From the complete data, six of 210 proteins were finally selected as suitable for LFQ normalization based on the following criteria: (1) their plasma concentrations remained nearly constant in all samples, as determined by their NormFinder stability value [33]; (2) their plasma concentrations did not differ significantly in the five responders and five non-responders, as shown by LMM analysis (*p*-value > 0.05); and (3) there were no reports of depression. The raw abundance of the six selected normalizing proteins, BTD, C8B, C1S, ITIH2, IGFALS, and SERPINA3, in each sample was divided by the geometric mean of six raw abundances in all samples. The median of these six ratios in a sample was defined as the normalization scaling factor (NSF) for that sample. The NSF for sample *s* can be calculated using the following equation:

$$NSF_{s}=geomean\left(\frac{N_{1,s}}{{\hat{N}}_{1}},\;\frac{N_{2,s}}{{\hat{N}}_{2}},\ldots ,\;\frac{N_{6,s}}{{\hat{N}}_{6}}\right)$$

where $N_{i,s}$ is the raw protein abundance of a normalization protein *i* in sample *s*, and ${\hat{N}}_{i}$ is the median abundance of protein *i* in all the samples. The normalized abundance of the intensity of each biomarker candidate in a sample was calculated by dividing its raw peak intensity by the NSF:

$$\overset{˘}{PA_{j,s}}=\frac{PA_{j.s}}{NSF_{s}}$$

where $\overset{˘}{PA_{j,s}}$ is the normalized abundance of the *j*-th biomarker candidate in sample *s*, and $PA_{j,s}$ is the raw abundance of the corresponding protein.

### 2.8. LC-ESI-MRM/MS Analysis

Liquid chromatography (LC) was performed on an Agilent 1290 Infinity UHPLC System with a reverse-phase ultra-high-performance LC (UHPLC) column (Agilent ZORBAX Eclipse Plus C18 Column, 95 Å, 2.1 mm i.d. × 100 mm, packed with 1.8 μm C18 resin) at a temperature of 50 °C. The mobile phases used in this study were 0.1% formic acid in water (solvent A) and 0.1% formic acid in acetonitrile (solvent B). The column was developed using a gradient of 0–2% solvent B for 5 min, 2–3% solvent B for 5 min, 3–50% solvent B for 10 min, 50–50% solvent B for 4 min, 50–0% solvent B for 1 min, and 0–0% solvent B for 9 min at a flow rate of 0.3 mL/min. The injected sample consisted of a mixture of digested plasma peptides (initial plasma volume: 40 μL) and isotope-labeled internal standard peptides. The UHPLC system was coupled to a triple quadrupole mass spectrometer (Agilent 6495 QQQ) by a standard-flow Jet Stream electrospray source operated in positive ion mode. Additional parameters included capillary voltage, 3.5 kV; nozzle voltage, 1 kV; gas temperature, 290 °C; drying gas flow rate, 11 L/min at 350 °C; nebulizer gas pressure, 40 PSI at 350 °C; and unit resolution for Q1 and Q3. MRM transitions were selected, and their collision energies optimized by Skyline (64-bit, version 19.1.0.193) software (Supplementary Table S4). The cell accelerator voltage was set to 5 V. Quantification experiments were performed using dynamic MRM (delta retention time: 3 min), with a total cycle time of approximately 1.5 s. The mass spectrometer was operated with MassHunter software (version B.08.00, Agilent), which generated MRM/MS data (*.d). MRM results from extracted ion chromatograms were analyzed by Skyline and quantified relative to the corresponding stable isotope-labeled peptides (SpikeTides™; JPT Peptdie Technologies Berlin, Germany).

### 2.9. Statistical Analysis

Data were analyzed using RStudio (version 1.1.456) including R (version 3.6.0). Longitudinal plasma protein abundance was assessed by LMM analysis (lme4 package), with drug response (non-response or response), sampling time (baseline, 1 week, 4 weeks, and 10 weeks), and response/time interaction and technical replications as fixed variables, and individual patients nesting for fixed variables and individuals as random variables. In the GEE analysis (geesmv package), we merged plasma abundance as the median of two or three technical replicates and then analyzed drug response, sampling time, and drug/sampling time. The working correlation structure was set independently, and Gaussian estimation was performed.

Clustering analysis was based on median protein concentrations in each group (responders and non-responders) at the four time points, and t-stochastic neighbor embedding (t-SNE) [34] (perplexity = 2, theta = 0, and dims = 2) and affinity propagation (method = correlation symmetry matrix and Spearman) were computed using Rtsne and apcluster [35] packages, respectably. Other software packages included ggline for scatter plots and psygenet2r for mapping proteins on the psychiatric disorders gene association network (PsyGeNet) at database = ”ALL” [36]. To control type I error by multiple comparisons, we applied the Bayesian sequential goodness of fit metatest (SGoF) method of default option (alpha = 0.05, gamma = 0.05, P0 = 0.5, a0 = 1, b0 = 1) in the SGoF R package [37] for *p*-values of response/time interaction by LMM analysis and Benjamini–Hochberg procedure [38] for *p*-values of MRM paired analysis, and then, we calculated permutated *p*-values for correlation analysis [39].

### 2.10. Literature Search

We performed a literature search on PubTabor central [40] using the keywords “protein name” AND “major depressive disorder” and identified 48 plasma proteins (37 proteins showing significance for response/interaction term in LMM and 11 proteins that significantly correlate with two or more psychiatric indexes). As used here, PubTator Central (PTC) is an online-based web page that automatically annotates the association between genes and diseases in PubMed abstracts and PMC full-text articles.

## 3. Results

### 3.1. Demographic and Clinical Characteristics of Study Subjects

The baseline characteristics of the ten study subjects, five responders, and five non-responders, are summarized in Table 1. Mean (standard deviation (SD)) subject age was similar in responders (44.2 (14.2) years) and non-responders (42.8 (16.4) years). There were no significant differences between the two groups in affective symptoms and disease severity, including their scores on the Montgomery and Asberg Depression Rating Scale (MADRS), the Clinical Global Impression-Severity (CGI-S), Beck’s Depression Inventory (BDI), the Hamilton Rating Scale for Depression (HAM-D), the Clinically Useful Depression Outcome Scale (CUDOS), and the World Health Organization Quality of Life abbreviated version scores including physical, psychological, social, and environmental quality of life (Supplementary Table S1).

### 3.2. Plasma Sample Preparations and Development of LC-MS/MS

Four plasma samples were obtained from each of the ten patients, for a total of 40 samples, and their proteins profiled by LC-MS/MS, with each sample assayed in duplicate or triplicate. A total of 1159 proteins were identified, with 684 proteins quantified by more than half and 206 proteins completely quantified in 104 of the LC-MS/MS measurements (Supplementary Table S2). Before comparing plasma protein abundance by the label-free quantification (LFQ) method, six relatively stable and abundant endogenous proteins (BTD, C8B, C1S, ITIH2, IGFALS, and SERPINA3) were chosen for data normalization of the abundance of other proteins, as described in the Materials and Methods section [41,42]. Following normalization of protein abundances in all experiments, sample-to-sample variations were corrected (Supplementary Figure S1A–C). Normalized abundances showed statistically significant correlations with the concentrations of plasma proteins (ng/mL) in the plasma proteome database [43], with a Pearson’s correlation coefficient of 0.677 (adjusted *p-*value < 0.001; Supplementary Figure S1D). Assuming technical variations were exceedingly small, only 346 detected proteins measured at one time in each individual sample were considered, followed by the elimination of 24 proteins associated with plasma depletion, including 14 plasma depletion target proteins and ten immunoglobulin-related proteins, and six normalization factors. A total of 316 proteins were analyzed in the next step, with missing values determined by a least-squares regression approach (Supplementary Table S3) [31,44].

### 3.3. Time-Dependent Changes in Plasma Proteins in Responders and Non-Responders

**Figure 1:**
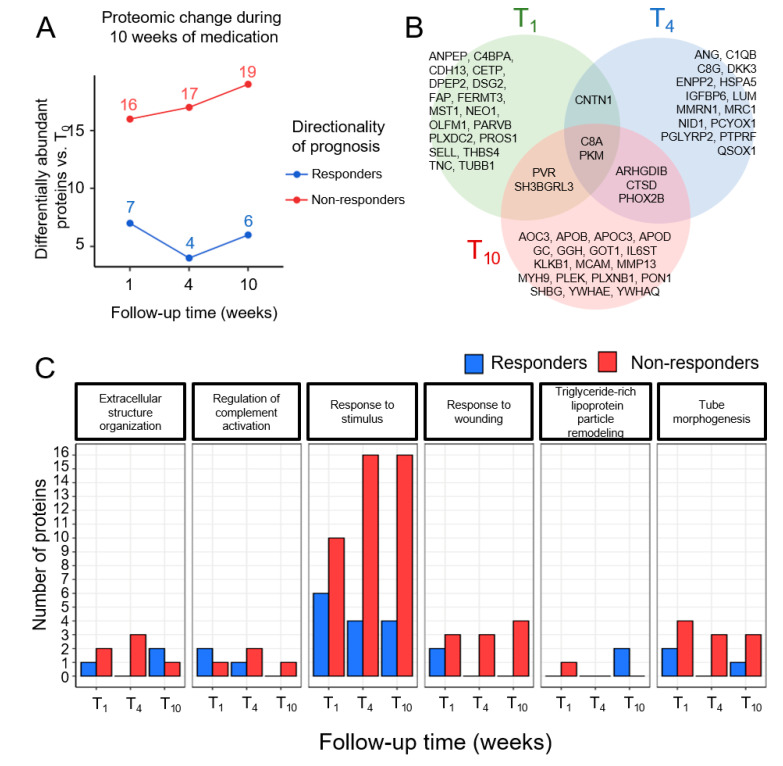
Plasma proteomic analyses and functional annotations identifying changes in differentially abundant proteins over the first week of drug administration. (**A**) Time-dependent up- and downregulation of differentially abundant proteins compared with the start of drug administration; T_0_. The number of proteins altered at each time point is shown above each time point. (**B**) Venn diagram of proteins differentially abundant at T_1_, T_4_, and T_10_ vs. T_0_. (**C**) Gene ontology terms of proteins differentially up- and downregulated at T_1_, T_4_, and T_10_ vs. T_0_.

Statistical comparisons of paired plasma protein abundances at baseline and after 1, 4, and 10 weeks of treatment showed that seven, four, and six proteins, respectively, were upregulated in responders and 16, 17, and 10 proteins, respectively, were upregulated in non-responders using the Mann–Whitney test without correction (*p*-value < 0.05; Figure 1A). The Venn diagram of the three time points, T_1_, T_4_, and T_10_ is shown in Figure 1B. Proteins upregulated in non-responders were associated with responses to wounding and stimuli, responses to wounding, and tube morphogenesis in the gene ontology (GO) biological process (Figure 1C). These findings may reflect the greater number of active inflammatory pathways with neural circuits of the brain in non-responders [45]. Proteins that fit the GO terms extracellular structure organization, regulation of complement activation, and triglyceride-rich lipoprotein particle remodeling were enriched in both groups.

LMM is appropriate for identifying differentially abundant plasma proteins based on longitudinal proteome data. The response/time interaction term is important in measuring inter-group differences in time-dependent responsiveness to SSRIs. Through the LMM multiple comparison analysis, we identified 37 significant proteins which were corrected by a SGoF method [37] (adjusted *p*-value < 0.05; response/time interaction term). These proteins over time, as well as the between-group differences, are shown as lowest adjusted *p*-values in Table 2.

**Figure 2:**
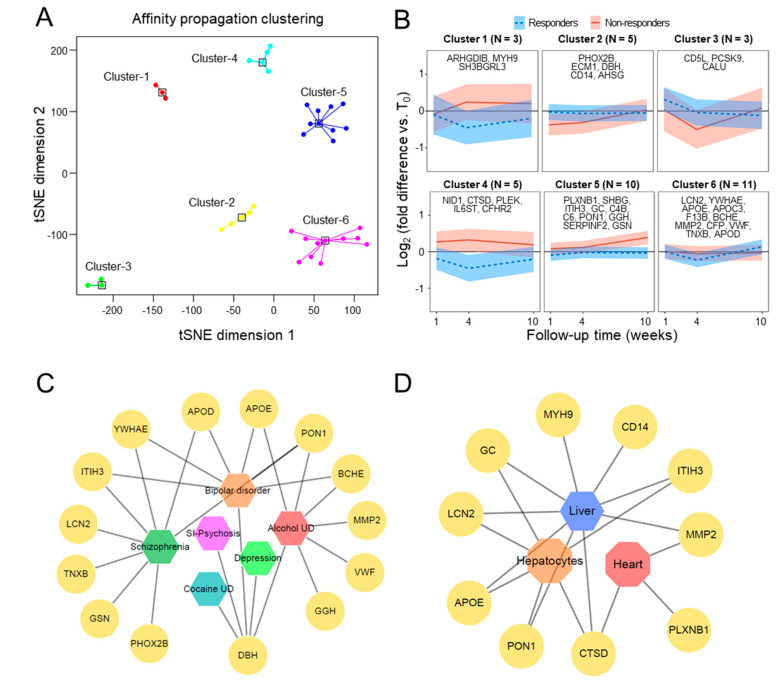
Affinity propagation clustering, profile analysis, and public database search of the 37 proteins found to differ significantly in the response/time interaction of linear mixed model (LMM). (**A**) Identification of seven protein clusters by t-SNE-based affinity propagation clustering. (**B**) Change over time in protein amount in responders and non-responders. (**C**) Association of 14 proteins found on PsyGeNet with psychiatric diseases. (**D**) Association of ten proteins found in the DrugMatrix category of Enrichr with responses of rat tissues and cells to selective serotonin reuptake inhibitors (SSRIs).

To better understand the abundance patterns and to cluster proteins with similar patterns, protein abundance at three different times (T_1_, T_4_, and T_10_) was subtracted from that at baseline (T_0_), followed by t-SNE and affinity propagation (Figure 2A). Six clusters of unique patterns were obtained (Figure 2B). The three and five proteins in clusters 1 and 4, respectively, decreased over time in responders and increased over time in non-responders. Cluster 2, which included five proteins, showed little change over time in responders but decreased over time in non-responders. In cluster 3, three proteins showed increase from week 1 and appeared flat week 4 onward in responders; conversely, the proteins showed a sharp decrease at week 4 and then flattened at week 10 in non-responders. In cluster 5, 10 proteins showed little change over time in responders but increased over time in non-responders. In cluster 6, 11 proteins showed decreases at 4 weeks and increases at 10 weeks in responders but little change over time in non-responders. The individual abundance profiles of the 37 proteins are shown in Supplementary Figure S2.

To assess whether the functional roles of these proteins were associated with antidepressant response and psychiatric disorders, we searched for the 37 proteins in the PsyGeNet (Figure 2C) [36]. APOD, APOE, BCHE, DBH, GGH, GSN, ITIH3, LCN2, MMP2, PHOX2B, PON1, TNXB, VWF, YWHAE, 14 of these proteins were found to be associated with psychiatric symptoms, such as schizophrenia, bipolar disorder, cocaine use disorders, substance-induced psychosis, alcohol use disorders, and depression. In addition, we assessed whether these 37 proteins were associated with citalopram, an analog of escitalopram, by searching responses of rat tissues and cells to SSRIs in the DrugMatrix category of Enrichr [46], a web-based gene enrichment analysis tool. We found that expression of nine proteins, ITIH3, PON1, MMP2, MYH9, APOE, GC, CD14, LCN2, and CTSD, differed significantly in SSRI-treated and control, corn oil-treated rat liver; the expression of seven proteins, ITIH3, PON1, LCN2, APOE, GC, CLU, and CTSD, differed significantly in SSRI- and corn oil-treated rat hepatocytes; and three proteins, PLXNB1, MMP2, and CTSD, differed significantly in SSRI- and corn oil-treated rat hearts (Figure 2D). It indicated that the drug reaction of these proteins causes quantitative changes not only in the blood but also in the organs of the liver and heart.

### 3.4. External Validation in Public Studies of mRNA Expression

**Figure 3:**
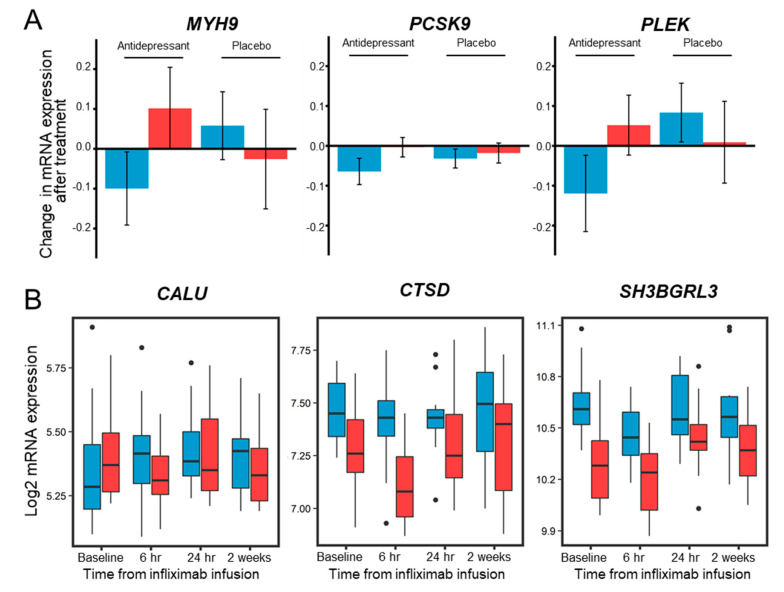
External validation of LMM-significant 37 proteins in two public GEO datasets (GSE146446 and GSE45468). (**A**) In the GSE146446 dataset, the quantitative mRNA expression changes in three genes, *MYH9*, *PCSK9*, and *PLEK*, before and after 8 weeks of taking the antidepressant and placebo in responders (blue color) and non-responders (red color). Error bars represent standard error of the mean. (**B**) In the GSE45468 dataset, the mRNA expression level of patients for three genes, *CALU*, *CTSD* and *SH3BGRL3*, before infliximab infusion and after 6 h, 24 h, and 2 weeks is shown in box plots. Responders are shown in blue color and non-responders are shown in red color.

Because we could not find a benchmark study on blood protein-based drug responsiveness to antidepressants, we examined the expression patterns of LMM-significant 37 proteins described in the results of large-scale studies at the blood circulating cell-free mRNA level from two publicly available GEO datasets—(GSE146446 [26] and GSE45468 [27]). Unlike the proteomic study above, the two GEO studies contained results on the effects of patients receiving a placebo. In the first GSE146446 dataset, mRNA expression in the blood of 171 depressed patients was studied, and patients’ responses to an antidepressant vs. the placebo were monitored. The antidepressant used was duloxetine. These data contain quantitative mRNA expressions in patients before and after 8 weeks of taking the antidepressant and placebo. There were 96 patients who received the drug, including 75 responders and 21 non-responders; and 107 patients received the placebo, including 44 responders and 63 non-responders. The 37 plasma proteins that were significant in time and response were all found in the dataset, and these were analyzed by LMM. Among them, *MYH9* represented significance for the treatment/response/time interaction term, *PCSK9* showed significance for the treatment/response interaction term (*p*-value < 0.05), and *PLEK* showed significance for treatment/response and treatment/time/response interaction terms (*p*-value < 0.05; Figure 3A). The second GSE45468 dataset reflected blood mRNA expression in 52 patients. These data included mRNA expression in patients before and after 6 h, 24 h, and 2 weeks of infusion of infliximab and a placebo. There were 23 patients who received the drug, including 12 responders and 11 non-responders, and there were 15 responders and 14 non-responders among 29 patients who received the placebo. In this dataset, only 13 out of 37 proteins were found and subjected to LMM analysis. Among them, *CALU* represented significance for the treatment/time/response interaction term (*p*-value < 0.05), and *CTSD* and *SH3BGRL3* represented significance for the treatment/response interaction term (*p*-value < 0.05; Figure 3B).

### 3.5. LC-MRM/MS Validation of Candidate Plasma Proteins Predictive of Early Response

**Figure 4:**
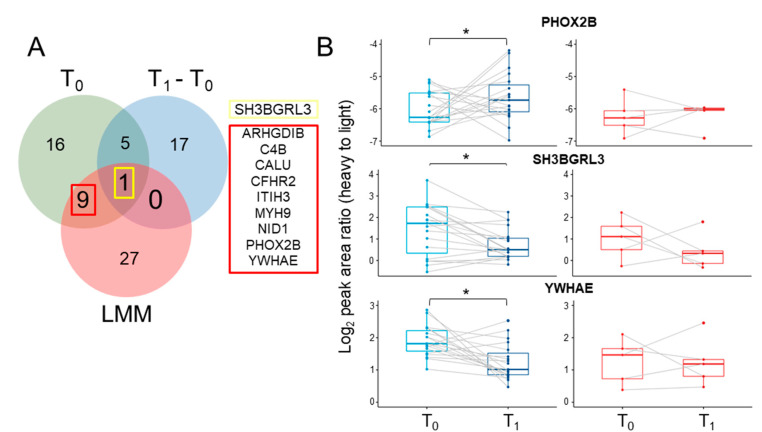
Liquid chromatography-multiple reaction monitoring/mass spectrometry (LC-MRM/MS) validation of ten candidate predictive biomarkers of early-drug response. (**A**) Venn diagram for detection of candidate biomarkers by three statistical analyses (T_0_ vs. T_1_ and T_1_–T_0_ between two groups, and response/time terms in LMM). (**B**) Boxplot of abundance of PHOX2B, SH3BGRL3, and YWHAE at T_1_ vs. T_0_, as determined by LC-MRM/MS, in responders (blue color) and non-responders (red color). * The asterisk identifies the adjusted *p*-values that are significant at the 0.05 level.

The differentially abundant ten proteins that were commonly significant between two groups at baseline, early treatment phase (from baseline to 1 week; Mann–Whitney U test: *p*-value < 0.05) and on the response/time interaction in LMM were selected (Figure 4A) and validated by serial isotope dilute-MRM/MS [47]. Among them, surrogate peptides were chosen by criteria except for CFHR2, which had no reliable peptide [48,49].

Based on reverse standard calibration curves (Supplementary Figure S3), 15 surrogate peptides representing nine proteins were selected for protein quantification, and a representative peptide with a strong signal for each protein was selected based on the LC-MRM/MS results (Supplementary Table S4). In the validation MRM result, comparing 19 responders and five non-responders, the MYH9 could not be quantified because the heavy-light ratio was below the limit of quantitation. Of the remaining eight proteins, the three proteins, PHOX2B, SH3BGRL3, and YWHAE, showed significant differences on baseline and week 1 in responders (Wilcoxon signed-rank test: FDR-adjusted *p*-value < 0.05) but not in non-responders (Wilcoxon signed-rank test: FDR-adjusted *p*-value > 0.05; Figure 4B). After 1 week, PHOX2B protein levels increased significantly, whereas SH3BGRL3 and YWHAE protein levels decreased significantly in responders; conversely, the three proteins did not show any significant changes in non-responders. By contrast, the level of the other five proteins did not differ significantly in the two groups (Supplementary Figure S4).

### 3.6. Relationship between Plasma Proteins and Psychiatric Morbidity Survey Scores

**Figure 5:**
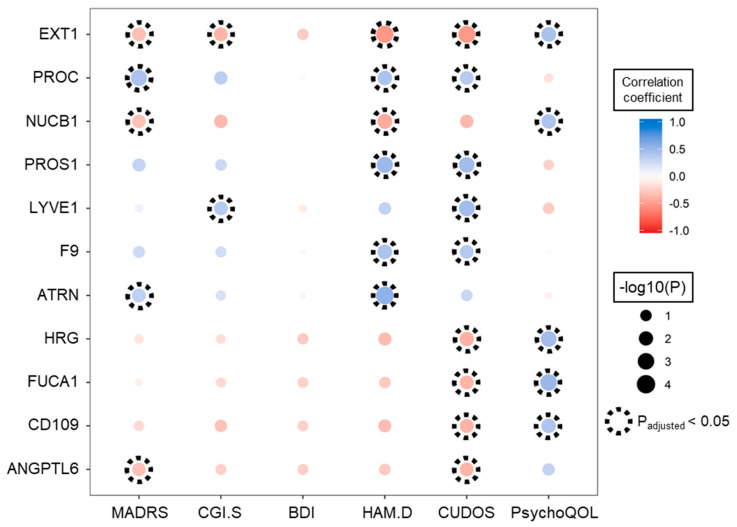
Plots showing correlations between the abundance of 11 plasma proteins and psychological indices; these 11 proteins correlated significantly with two or more of the six indices. Positive and negative correlation coefficients are colored blue and red, respectively, with the size of the bubble indicating the minus log10 adjusted *p-*value, and the dotted circles indicating significant correlation relationships (adjusted *p*-value < 0.05).

Because MADRS score is the standard criterion for determining response to drug administration, plasma proteins with high positive or negative correlation with MADRS scores indirectly reflect the efficacy of the drug. Using Spearman’s correlation analysis of 316 quantified proteins, we determined the significant correlation relationship between the abundance of the 64 identified plasma proteins and at least one other psychiatric index, such as CGI-S, BDI, HAM-D, CUDOS, and psychological quality of life (PsychoQOL) scores by permutation-based analysis (Supplementary Table S5). Each of these 11 proteins, EXT1, PROC, NUCB1, PROS1, LYVE1, F9, ATRN, HRG, FUCA1, CD109 and ANGPTL6, significantly correlated with two or more of the psychiatric indices (adjusted *p*-value < 0.05; Figure 5).

## 4. Discussion

In this pilot study, longitudinal analysis with a small sample size (N = 10) may estimate biased variation and cause inflation of type I error. Statistical techniques using a small sample number have been developed in psychiatry for circumstances wherein sample collection is not easy [50]. To discuss this part, we built a GEE model in addition to the LMM model. Compared to LMM, GEE showed greater statistical validity with a devised variance estimate even in a small number of samples [18]. We applied one of them, the Wang and Long method [51], modified bias-correction and efficiency improvement. Consequently, we found seven significant proteins for the time/response interaction term (adjusted *p*-value < 0.05; Wald test and corrected by SGoF; Supplementary Table S6), overlapping with five (AHSG, IL6ST, APOD, PHOX2B, and SHBG) of 37 significant proteins found in LMM. Unlike a GEE, which is a population level-based model and relatively easy to compute, LMM can consider random effects with technical or biological variation obtained from the whole data, and thus, we considered the LMM technique more suitable than GEEs [15,50,52].

Regarding protein biomarker candidates, we identified 37 plasma proteins significantly associated with MDD by the LMM analysis, and these protein biomarkers could be biologically or physiologically divided into four functional categories through the literature search on PubTabor central [40]. First, six plasma markers, GC, LCN2, ITIH3, VWF, PHOX2B and YWHAE, were previously reported to be associated with SSRI efficacy, with the abundances of GC, LCN2, and ITIH3 in plasma samples associated with response to SSRIs [21,53,54,55]. Among them, PHOX2B and YWHAE were validated by LC-MRM/MS analysis in this study. SSRI stress in human brain cells was reported to be associated with a PHOX2B transcription factor [56]. The YWHAE genes have been reported to play a significant role in MDD in the Han Chinese population, with alterations in their protein–protein interactions [57]. The second category is that sex hormones, neurotransmitters, and related proteins have been strongly associated with depression [58]. In this study, plasma SHBG, which has been previously linked to depression [59,60,61], showed the sharpest difference over time between responders and non-responders (adjusted *p*-value = 2.70 × 10^-3^), decreasing gradually with time in responders and increasing gradually with time in non-responders. We also found that plasma dopamine beta-hydroxylase (DBH) concentrations increased in responders from 4 to 10 weeks, consistent with low plasma DBH levels associated with low activity of the noradrenaline system in patients with depression [62,63,64,65,66]. Third, we found that the plasma proteins GSN and C4B were biomarkers of depression, similar to findings in previous studies using the same LC-MS platform [67,68,69]. The level of C4B was significantly higher in responders than in non-responders and showed a significant change over time. Subsequently, APOD, PON1, BCHE, and IL6ST were reported to be related to depression, a finding consistent with our results [21,70,71,72,73,74]. Finally, APOE, CSTD and MMP2 that varied genetically and in mRNA level of abundance were reported to be associated with depression. We found that the levels of abundance of two of these proteins, APOE and CSTD, with single nucleotide polymorphisms (SNPs) differed significantly in responders and non-responders [75,76,77,78]. The expression of the MMP2 gene in the brain was associated with recurrence of depression [55].

Moreover, 11 plasma proteins that strongly correlated with two or more psychiatric indexes were related with neurological mechanisms and SSRI response. EXT1 was involved in the biosynthesis of heparan sulfate, which played an important role in the development of the nervous systems in the brain, and its deletion caused autism-like behavior in mice [79,80]. NUCB1 is known as a Golgi-resident marker of neurons [81] and interrupts amyloid fibrillation in the brain [82]. PROS1 turned out to be a novel Aβ-responsive protein based on proteome profiling of the hippocampus in the 5XFAD mouse model [83]. LYVE1 was the upregulated gene expressed in SSRI responders to non-responders [84], and differential LYVE1 and MHC II expression was used to identify CNS border-associated macrophages in single cell experiments [85]. ATRN, a neuroprotectant [86], was high in the SSRI responder in blood proteins. CD109 was higher in the disease group in the plasma proteome comparison between the psychotic disorder and the normal group [87].

This preliminary retrospective study had several limitations, including its small sample size, the lack of racial diversity among the study subjects, and the collection of plasma samples at a single center. Proteomics studies using small specimens are frequent. Typically, 10–50 samples are used during the preclinical discovery and validation phase, given the analysis of large data sets and limited timelines [88]. Thus, our results should be considered preliminary findings. All participants were Korean population, and our results may not be generalizable to other ethnic groups. In addition, plasma protein abundance may be affected by the plasma preparation method [89,90], but plasma was rapidly prepared from blood and stored frozen at −80 °C to avoid any pre-analytical effect [91,92,93]. Moreover, alterations in plasma protein abundance may be dependent on the SSRI type and dosage. In this study, we used samples treated with the same antidepressant (escitalopram) in a relatively certain range of doses, and this may be a limitation in using the results of this study to predict treatment responses with other antidepressants. However, this can be considered the strength of this study. As it is clinically difficult to collect plasma samples using the same type and dose of antidepressants for patients with major depressive disorders in a prospective design, so far, most studies on protein biomarkers for antidepressant treatment response have not been able to control the types or doses of antidepressants [69]. In the view of personalized treatment, predicting whether an individual with depression will benefit from a particular antidepressant is critical in choosing the right antidepressant; furthermore, how different types of antidepressants affect plasma proteins should be considered. In this study, the use of samples treated with the same antidepressant (escitalopram) in a relatively certain range of doses is considered a strength of this study. A controlled prospective study with a large sample size is necessary to establish a clear differential influence of several types of antidepressants on plasma proteins. Therefore, prospective studies in larger patient cohorts are needed to validate our findings.

## 5. Conclusions

To monitor the association between the efficacy of SSRIs and biomarker abundance, plasma samples were collected for 10 weeks during treatment of patients with MDD. Biomarkers have been identified through longitudinal measurements of protein concentrations, with some showing significant correlation with mental disease variables. These findings suggest that the liquid biopsy technique may solve unmet clinical problems.

## Figures and Tables

**Table 1:** Demographic and clinical variables of study subjects.

Variable	Responders (N = 5)	Non-Responders (N = 5)	*p*-Value
Age (SD)	44.2 (14.2)	42.8 (16.4)	0.841
Male (%)	1 (20)	1 (20)	1.000
Age at onset (SD)	41.8 (11.9)	33.4 (9.8)	0.093
Body mass index (kg/m^2^) (SD)	23.1 (3.7)	24.7 (4.6)	0.309
Clinical characteristics at baseline			
Montgomery and Asberg Depression Rating Scale (SD)	31.0 (4.6)	28.8 (2.5)	0.599
Clinical Global Impression-Severity (SD)	5.0 (0.7)	4.2 (1.3)	0.344
Beck’s Depression Inventory (SD)	32.6 (7.3)	26.8 (3.6)	0.206
Hamilton Rating Scale for Depression (SD)	21.6 (3.4)	21.0 (2.9)	1.000
Clinically Useful Depression Outcome Scale (SD)	38.4 (12.8)	40.4 (3.0)	0.917
World Health Organization Quality of Life abbreviated version			
Physical quality of life (SD)	8.8 (1.7)	8.5 (0.9)	0.831
Psychological quality of life (SD)	8.0 (0.8)	8.3 (1.7)	0.827
Social quality of life (SD)	10.1 (1.5)	11.7 (2.4)	0.193
Environmental quality of life (SD)	10.1 (1.7)	10.1 (1.1)	0.914

*p*-values appropriately calculated using the Mann–Whitney U test or Fisher’s exact test.

**Table 2:** 37 differentially abundant proteins corresponding to response/time interaction.

UNIPROT Accession	Adjusted *p*-Value	Gene Name	Protein Name	Cluster No.	COR ^a^
P04278	2.70 × 10^-3^	SHBG	Sex hormone-binding globulin	5	0.12
P05090	2.95 × 10^-3^	APOD	Apolipoprotein D	6	−0.34 ^b^
Q06033	4.01 × 10^-3^	ITIH3	Inter-alpha-trypsin inhibitor heavy chain H3	5	−0.21
P08567	4.36 × 10^-3^	PLEK	Pleckstrin	4	0.04
P04275	4.69 × 10^-3^	VWF	von Willebrand factor	6	−0.19
P52566	5.11 × 10^-3^	ARHGDIB	Rho GDP-dissociation inhibitor 2	1	−0.15
P02656	6.89 × 10^-3^	APOC3	Apolipoprotein C-III	6	−0.06
P06276	7.13 × 10^-3^	BCHE	Cholinesterase	6	−0.11
P27169	8.89 × 10^-3^	PON1	Serum paraoxonase/arylesterase 1	5	−0.08
P22105-4	9.85 × 10^-3^	TNXB	Tenascin-X	6	−0.25
P02774-3	1.09 × 10^-2^	GC	Vitamin D-binding protein	5	0.04
P0C0L5	1.10 × 10^-2^	C4B	Complement C4-B	5	−0.21
P02649	1.15 × 10^-2^	APOE	Apolipoprotein E	6	−0.04
P07339	1.45 × 10^-2^	CTSD	Cathepsin D	4	0.02
Q92820	1.50 × 10^-2^	GGH	Gamma-glutamyl hydrolase	5	−0.01
P09172	1.69 × 10^-2^	DBH	Dopamine beta-hydroxylase	2	0.03
P40189	1.75 × 10^-2^	IL6ST	Interleukin-6 receptor subunit beta	4	0.15
Q8NBP7	1.81 × 10^-2^	PCSK9	Proprotein convertase subtilisin/kexin type 9	3	−0.14
Q16610	1.83 × 10^-2^	ECM1	Extracellular matrix protein 1	2	0.02
P62258	1.83 × 10^-2^	YWHAE	14-3-3 protein epsilon	6	0.18
P80188	1.83 × 10^-2^	LCN2	Neutrophil gelatinase-associated lipocalin	6	−0.11
Q9H299	1.99 × 10^-2^	SH3BGRL3	SH3 domain-binding glutamic acid-rich-like protein 3	1	0.13
P27918	2.05 × 10^-2^	CFP	Properdin	6	0.08
P08571	2.12 × 10^-2^	CD14	Monocyte differentiation antigen CD14	2	0.24
P08697	2.33 × 10^-2^	SERPINF2	Alpha-2-antiplasmin	5	0.22
P36980	2.34 × 10^-2^	CFHR2	Complement factor H-related protein 2	4	0.16
P08253	2.57 × 10^-2^	MMP2	72 kDa type IV collagenase	6	0.13
P13671	2.65 × 10^-2^	C6	Complement component C6	5	0.13
O43852-3	2.80 × 10^-2^	CALU	Calumenin	3	0.10
P14543	2.93 × 10^-2^	NID1	Nidogen-1	4	−0.05
P35579	2.95 × 10^-2^	MYH9	Myosin-9	1	0.05
P05160	3.70 × 10^-2^	F13B	Coagulation factor XIII B chain	6	−0.17
P02765	3.75 × 10^-2^	AHSG	Alpha-2-HS-glycoprotein	2	0.20
Q99453	3.85 × 10^-2^	PHOX2B	Paired mesoderm homeobox protein 2B	2	−0.12
O43157	4.01 × 10^-2^	PLXNB1	Plexin-B1	5	−0.01
P06396	4.26 × 10^-2^	GSN	Gelsolin	6	0.04
O43866	4.41 × 10^-2^	CD5L	CD5 antigen-like	3	−0.27

^a^ Spearman’s correlation coefficient of protein abundance and Montgomery and Asberg Depression Rating Scale (MADRS) for each protein. ^b^ Adjusted *p*-values < 0.05 on a permutated correlation test based on Spearman’s coefficient analysis.

## Supplementary Materials

The following are available online at https://www.mdpi.com/2227-9059/8/11/455/s1: Figure S1. Box plots for protein abundances in each LC-MS/MS run. Protein abundances (a) before and (b) after endogenous protein-based normalization. (c) Two-dimensional global t-SNE map comparing the responders (blue) and non-responders (red) at each sampling time (1, 4, and 10 weeks) and 3 t-SNE parameters of perplexity. (d) Plasma protein log2 abundances (ng/mL) in the Plasma Proteome Database (bottom) and normalized protein abundance (right). Figure S2. Changes in responders over time to the amounts of 37 significant proteins belonging to six clusters, as determined by response/time interaction. Clusters 1 through 6 included 3, 5, 3, 5, 10 and 11 proteins, respectively. Figure S3. Calibration curves of 15 surrogate peptides relative to nine proteins based on heavy-to-light extracted ion chromatogram ratio. Figure S4. MRM results of five proteins at T_0_ vs. T_1._ Boxplot of five quantified proteins paired at T_0_ and T_1_ in responders and non-responders. Table S1. Demographic and clinical characteristics of the ten depressed patients at each of the four hospital visits. Table S2. The 1,159 plasma proteins identified in 104 LC-MS/MS measurements. The notation system is (sample name)_(response)_(time)_(experiment number). Table S3. Log2 transformed normalized protein abundance of 316 proteins in 104 LC-MS/MS measurements. Table S4. MRM parameters optimized for 14 target peptides of nine proteins. Table S5. Spearman’s rank correlation coefficients and adjusted *p-*values for the relationships between 316 protein abundance and six psychiatric symptom indices. Table S6. Estimation results in generalized estimation equation.

## Abbreviations

MDD	Major depressive disorder
SSRIs	Selective serotonin reuptake inhibitors
STAR*D	Sequenced Treatment Alternative to Relieve Depression
LC-MS/MS	Liquid chromatography tandem mass spectrometry
MEM	mixed-effect model
GEE	generalized estimating equation
LMM	Linear mixed model
LC-MRM/MS	Liquid chromatography-multiple reaction monitoring/mass spectrometry
LFQ	Label-free quantification
SD	Standard deviation
MADRS	Montgomery and Asberg Depression Rating Scale
CGI-S	Clinical Global Impression-Severity
BDI	Beck’s Depression Inventory
HAM-D	Hamilton Rating Scale for Depression
CUDOS	Clinically Useful Depression Outcome Scale
GO	Gene ontology
SGoF	Sequential goodness of fit metatest
t-SNE	t-stochastic neighbor embedding
PsyGeNet	Psychiatric disorders gene association network
PsychoQOL	Psychological quality of life
DBH	Dopamine beta-hydroxylase
NSF	Normalization scaling factor
