# Supplementary material for: An Exploratory Pilot Study with Plasma Protein Signatures Associated with Response of Patients with Depression to Antidepressant Treatment for 10 Weeks
Source: Biomedicines. 2020 Oct 28;8(11):455. doi: 10.3390/biomedicines8110455 (PMC7692261; doi:10.3390/biomedicines8110455)
Supplement: Supplementary file 1 [file biomedicines-08-00455-s001.zip › Supplementary Figure.pdf]

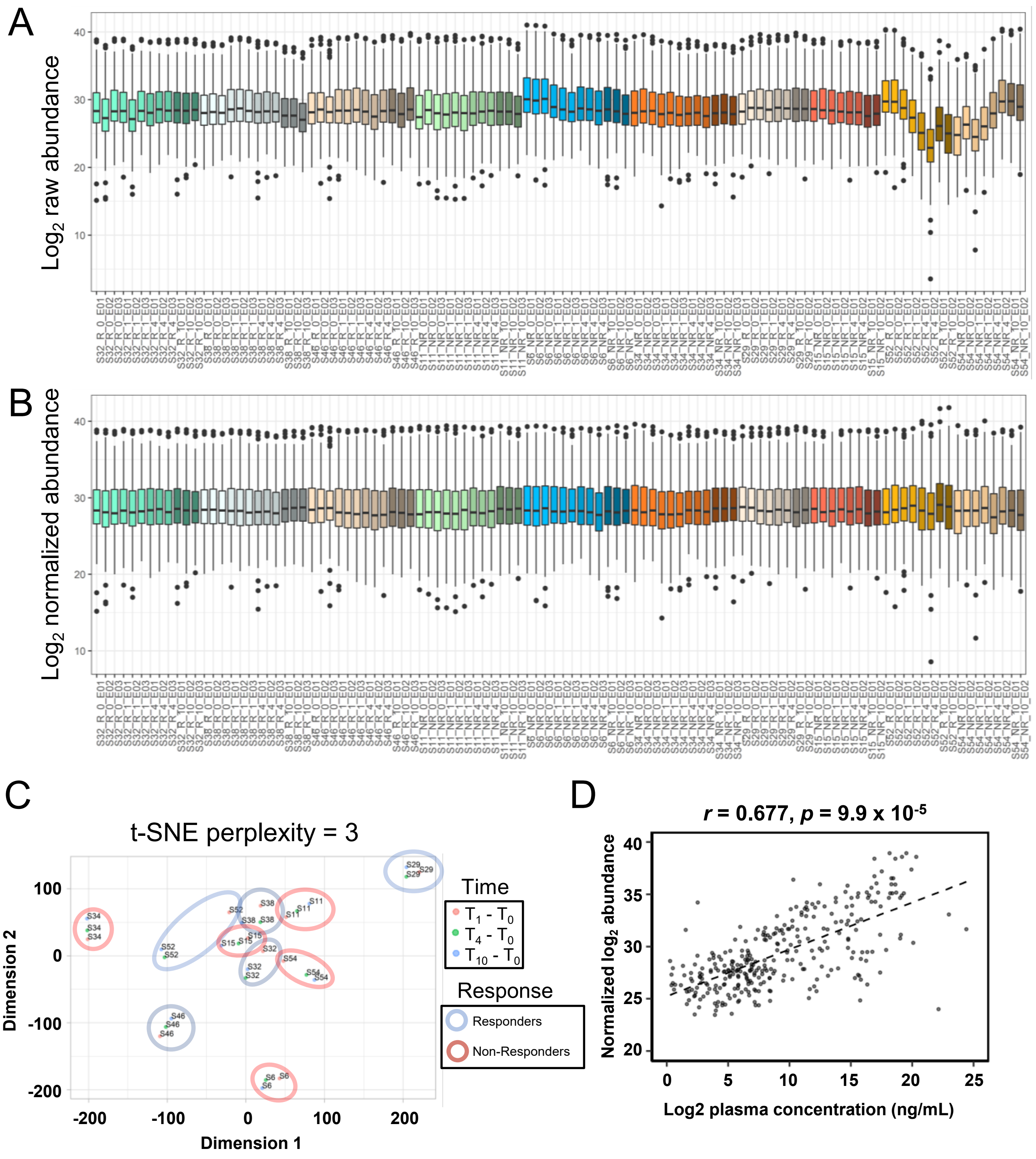

Figure S1: Box plots for protein abundances in each LC-MS/MS run. Protein abundances (**a**) before and (**b**) after endogenous protein-based normalization. (**c**) Two-dimensional global t-SNE map comparing the responders (blue) and non-responders (red) at each sampling time (1, 4, and 10 weeks) and 3 t-SNE parameters of perplexity. (**d**) Plasma protein log2 abundances (ng/mL) in the Plasma Proteome Database (bottom) and normalized protein abundance (right).

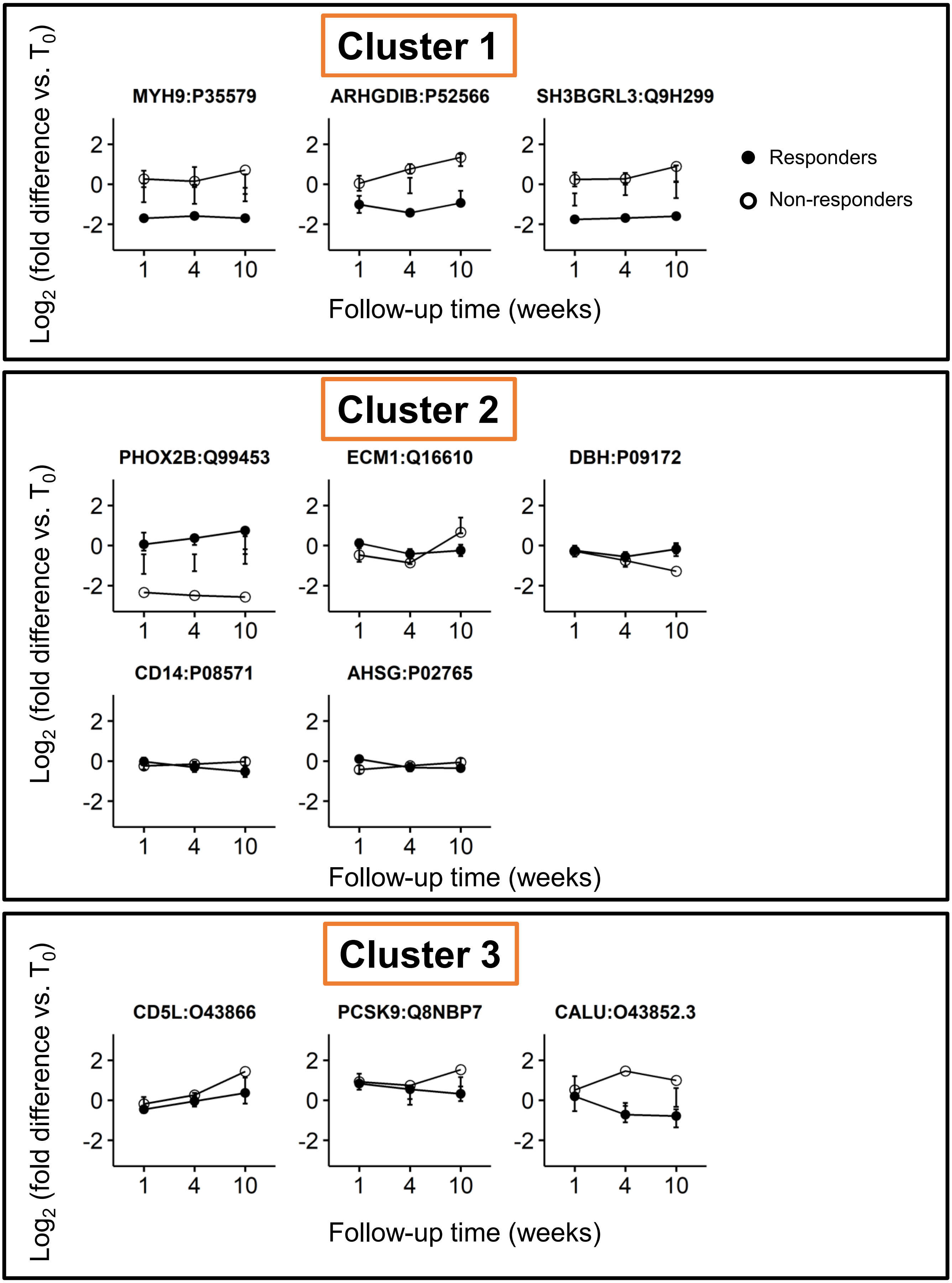
Cluster 1
MYH9:P35579
ARHGDIB:P52566
SH3BGRL3:Q9H299
Responders
Non-responders
Log2 (fold difference vs. T0)
Follow-up time (weeks)
Cluster 2
PHOX2B:Q99453
ECM1:Q16610
DBH:P09172
CD14:P08571
AHSG:P02765
Log2 (fold difference vs. T0)
Follow-up time (weeks)
Cluster 3
CD5L:O43866
PCSK9:Q8NBP7
CALU:O43852.3
Log2 (fold difference vs. T0)
Follow-up time (weeks)

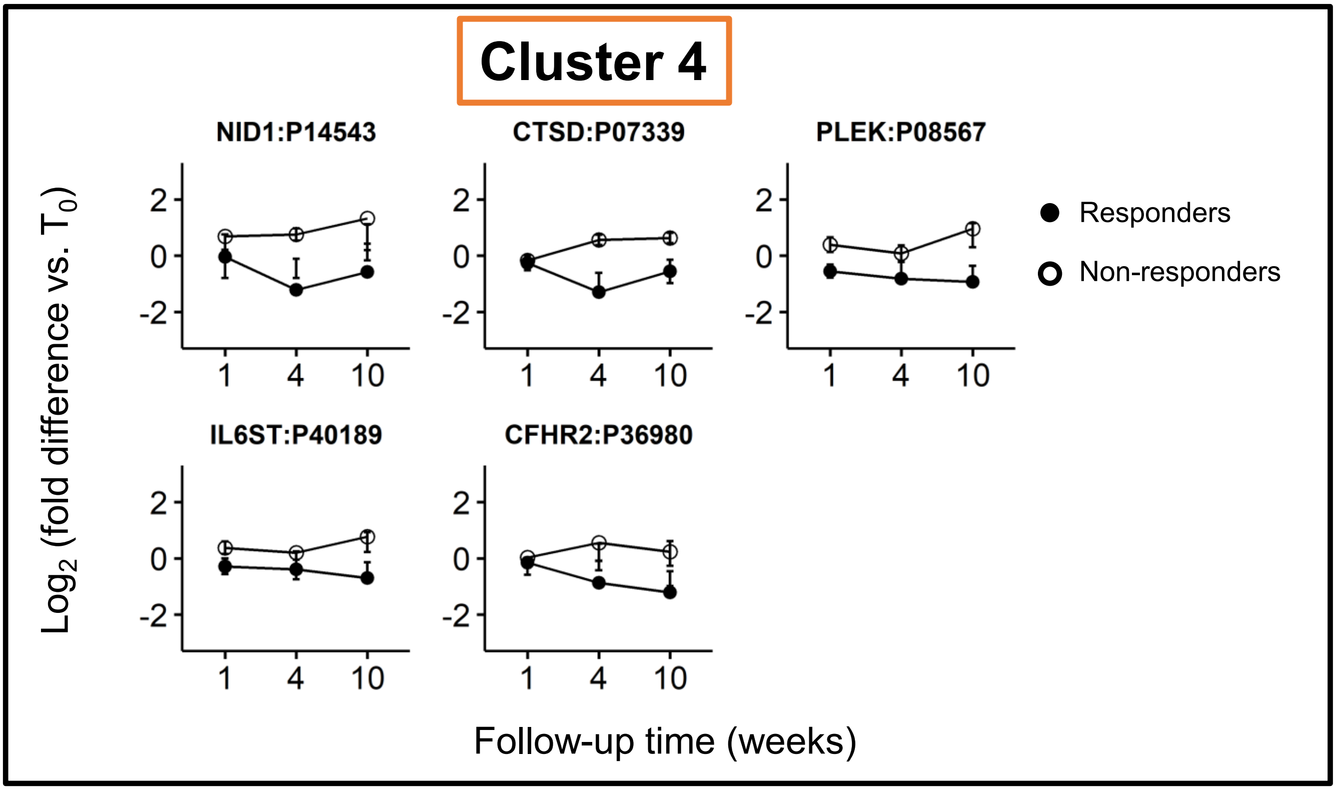
Cluster 4
NID1:P14543
CTSD:P07339
PLEK:P08567
IL6ST:P40189
CFHR2:P36980
Responders
Non-responders
Log2 (fold difference vs. T0)
Follow-up time (weeks)

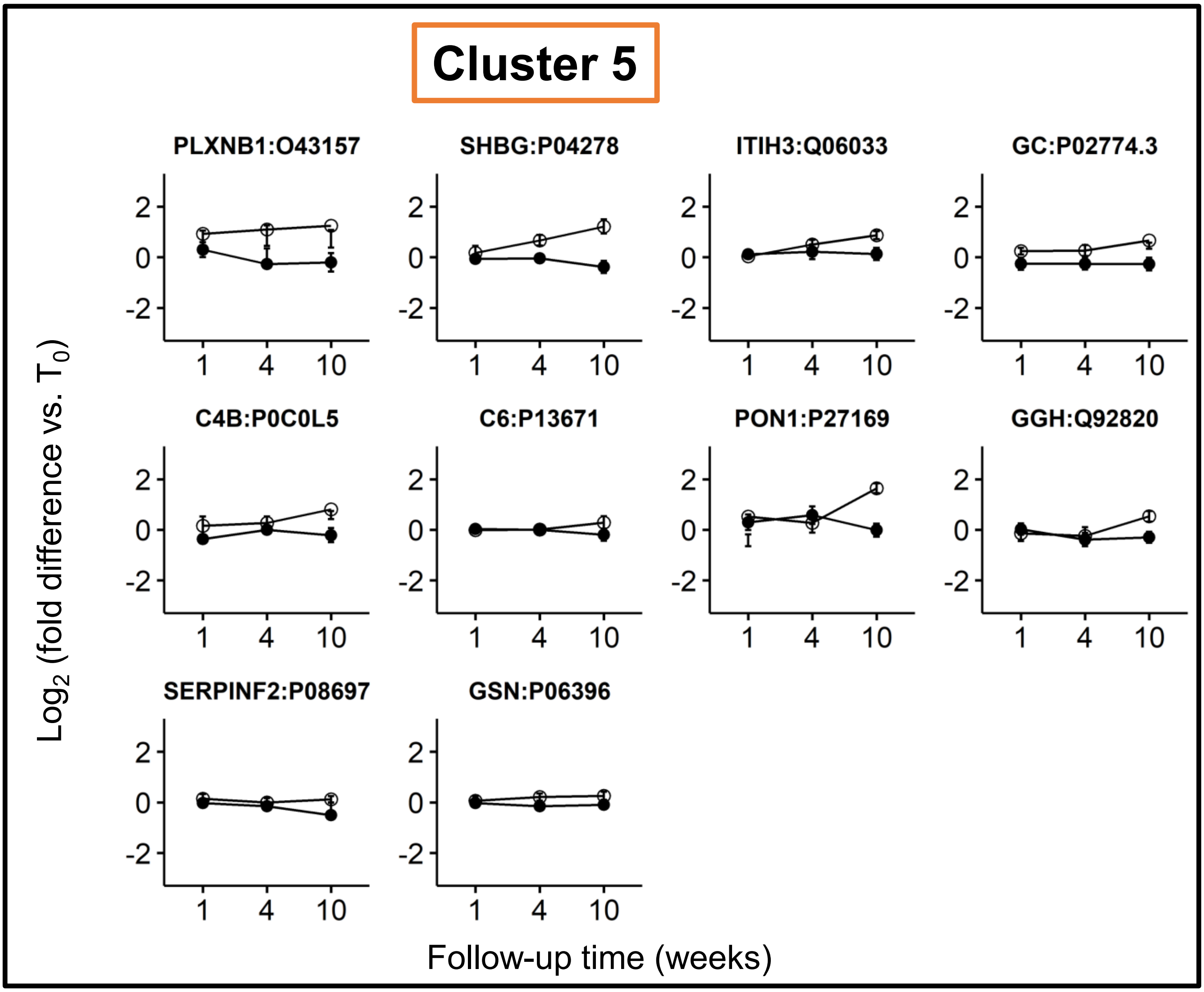
Cluster 5
PLXNB1:O43157
SHBG:P04278
ITIH3:Q06033
GC:P02774.3
C4B:P0C0L5
C6:P13671
PON1:P27169
GGH:Q92820
SERPINF2:P08697
GSN:P06396
Log2 (fold difference vs. T0)
Follow-up time (weeks)

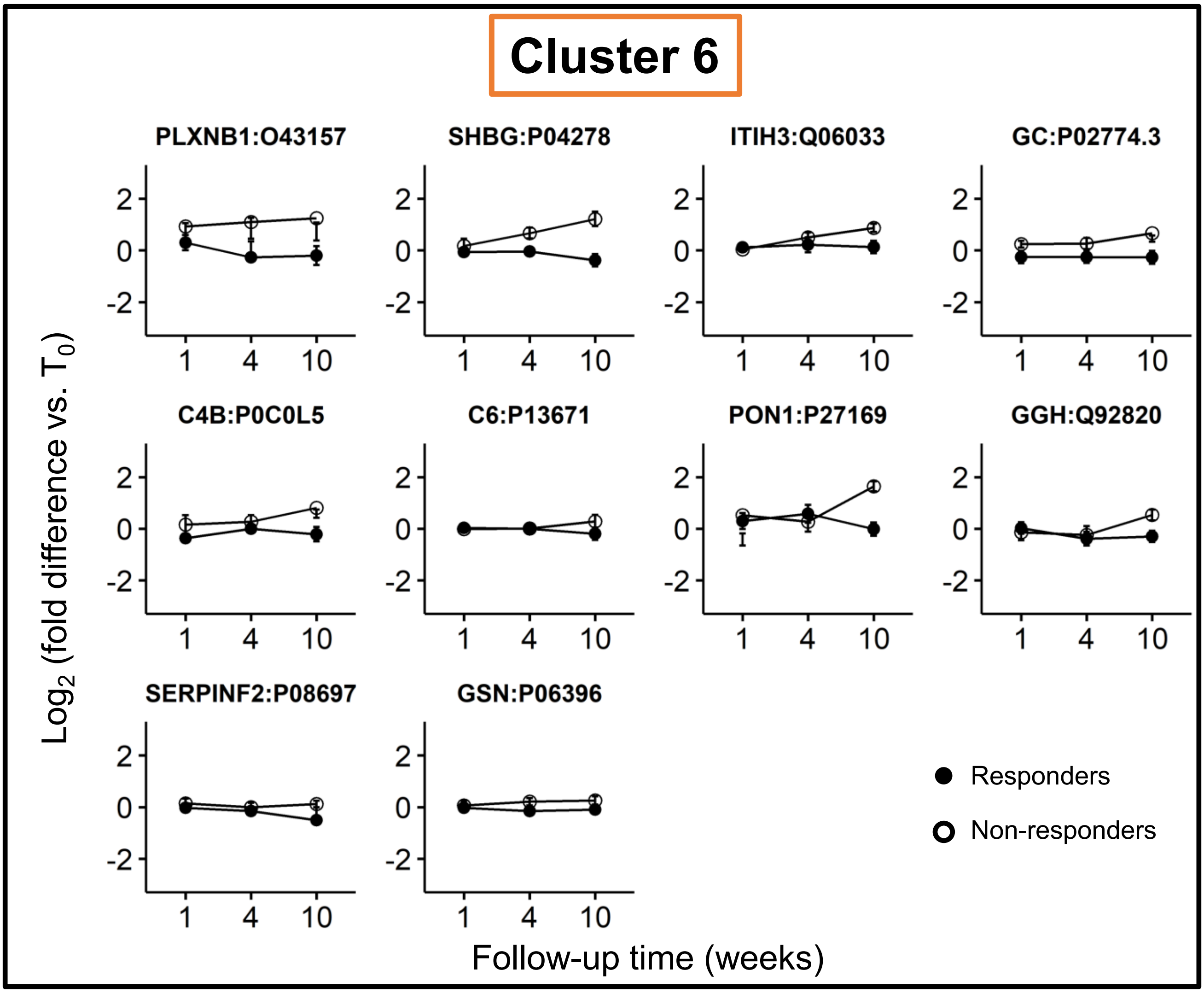

Figure S2: Changes in responders over time to the amounts of 37 significant proteins belonging to six clusters, as determined by response/time interaction. Clusters 1 through 6 included 3, 5, 3, 5, 10 and 11 proteins, respectively.

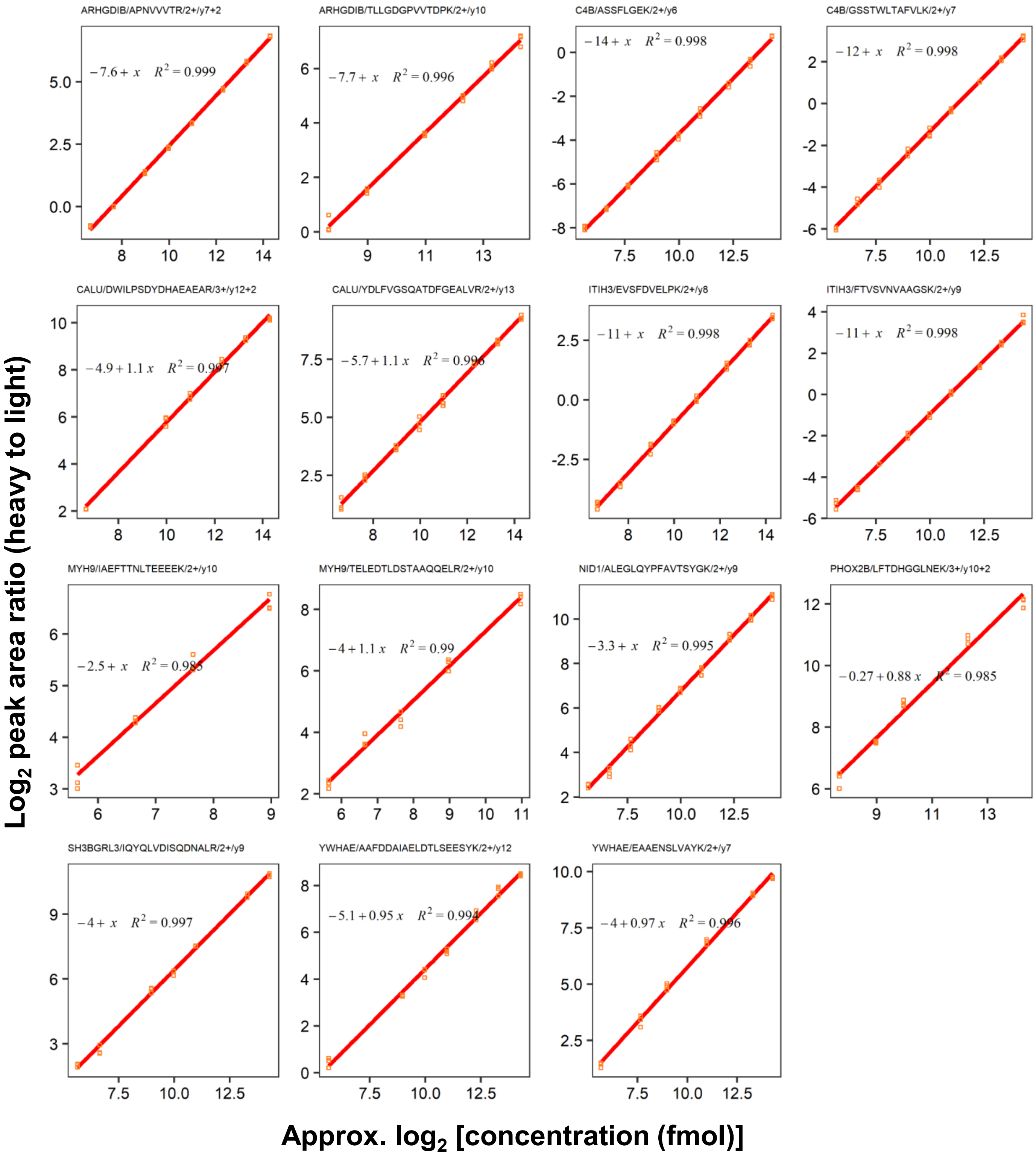

Figure S3: Calibration curves of 15 surrogate peptides relative to nine proteins based on heavy-to-light extracted ion chromatogram ratio.

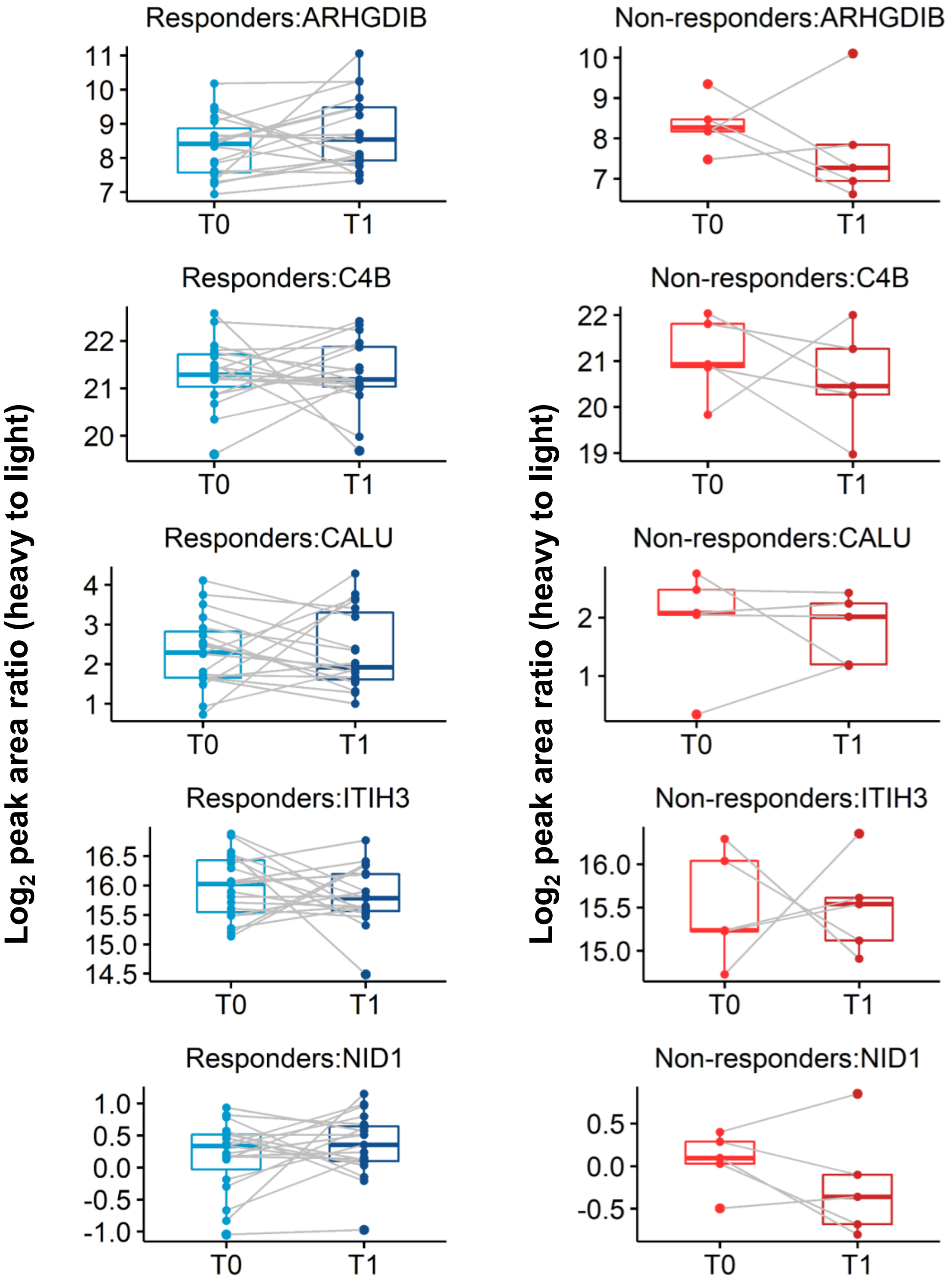

Figure S4: MRM results of five proteins at T_0 vs. $T_{1.}$ Boxplot of five quantified proteins paired at T_0 and T_1 in responders (blue color) and non-responders (red color).
